# *Ginkgo biloba* Prevents Oxidative Stress-Induced Apoptosis Blocking p53 Activation in Neuroblastoma Cells

**DOI:** 10.3390/antiox9040279

**Published:** 2020-03-26

**Authors:** Francesco Di Meo, Rossana Cuciniello, Sabrina Margarucci, Paolo Bergamo, Orsolina Petillo, Gianfranco Peluso, Stefania Filosa, Stefania Crispi

**Affiliations:** 1 Institute of Biosciences and BioResources-UOS Naples CNR, Via P. Castellino 111, 80131 Naples, Italy; francesco.dimeo.90@gmail.com (F.D.M.); rossanacuciniello@gmail.com (R.C.); 2 Department of Biology, University of Naples Federico II, Complesso Universitario Monte Sant’Angelo Via Cinthia, 80126 Naples, Italy; 3 Institute on Terrestrial Ecosystems (IRET) CNR, Via P. Castellino 111, 80131 Naples, Italy; sabrina.margarucci@cnr.it (S.M.); orsolina.petillo@cnr.it (O.P.); gianfranco.peluso@cnr.it (G.P.); 4 Institute of Food Science CNR, Via Roma, 64, 83100 Avellino, Italy; paolo.bergamo@isa.cnr.it; 5 IRCCS Neuromed, Localitá Camerelle, 86077 Pozzilli (IS), Italy

**Keywords:** *ginkgo biloba*, oxidative stress, p53, apoptosis, neuroprotection, neuroblastoma cells

## Abstract

Oxidative stress has been associated to neuronal cell loss in neurodegenerative diseases. Neurons are post-mitotic cells that are very sensitive to oxidative stress—especially considering their limited capacity to be replaced. Therefore, reduction of oxidative stress, and inhibiting apoptosis, will potentially prevent neurodegeneration. In this study, we investigated the neuroprotective effect of *Ginkgo biloba* extract (EGb 761) against H_2_O_2_ induced apoptosis in SK-N-BE neuroblastoma cells. We analysed the molecular signalling pathway involved in the apoptotic cell death. H_2_O_2_ induced an increased acetylation of p53 lysine 382, a reduction in mitochondrial membrane potential, an increased BAX/Bcl-2 ratio and consequently increased Poly (ADP-ribose) polymerase (PARP) cleavage. All these effects were blocked by EGb 761 treatment. Thus, EGb 761, acting as intracellular antioxidant, protects neuroblastoma cells against activation of p53 mediated pathway and intrinsic mitochondrial apoptosis. Our results suggest that EGb 761, protecting against oxidative-stress induced apoptotic cell death, could potentially be used as nutraceutical for the prevention and treatment of neurodegenerative diseases.

## 1. Introduction

Oxidative stress occurs in the cell when the antioxidant defence in unable to balance the rate of the reactive oxygen species generated [1]. The cellular response to oxidative stress mainly depends on the injury intensity and duration. Oxidant sources can be external to the cell, such as environmental stress, or they can be generated as a side product of normal aerobic metabolism. 

When present in excess, oxidants elevate the intracellular levels of reactive oxygen species (ROS) and damage cell membrane, proteins and DNA. Oxidative stress generates different consequences ranging from stimulation of cell proliferation to cell cycle arrest, stimulation or inhibition of cells migration, and up to cell death by apoptosis or necrosis [2,3,4]. 

Among different cell types, neurons are particularly prone to production of ROS and highly susceptible to redox stress, because of their high lipid and metal ion content combined with their high metabolic rate and relatively low concentrations of antioxidants [5]. Oxidative stress is considered a relevant direct or indirect process associated to neuronal cell loss in neurodegenerative diseases such as Parkinson’s disease [6] and Alzheimer’s disease [7]. Despite the differences in clinical manifestations, neurological disorders show common pathological processes. All of them are characterized by degeneration and progressive loss of distinct neuron subsets in specific areas of the brain. Moreover, they share common neurodegenerative pathways associated with progressive neuronal dysfunction. Most of these pathways seems to be related to oxidative stress, to the imbalance between generation of free radicals and cellular antioxidant defences, and to apoptotic cell death [8]. Neuronal cell death is especially dangerous because adult neurons are post-mitotic cells with limited capacity to proliferate or be replaced. Therefore, in neuronal cells, reduction of oxidative stress could inhibit apoptosis potentially preventing neurodegeneration.

Herbal extracts and phytochemicals can act as protective agent against oxidative stress. Flavonoids are in particular described as the molecules with a strong bioactivity in brain functions with positive effects on synaptic plasticity and neuronal activity. Among the bioactive phytochemicals, *Ginkgo biloba* is one of the most used worldwide [9]. *Ginkgo biloba* has been widely used in the treatment of cardiovascular and cerebrovascular diseases, liver cirrhosis and acute and chronic renal disease. More recently, a standardized extract of *Ginkgo biloba*, EGb 761, has been found to have neuroprotective effects in several central nervous system and neurodegenerative diseases [9]. 

EGb 761 (EGb) is a standardised extract of *Ginkgo biloba* leaves that contains a well-defined concentration of flavone glycosides and terpene lactones (24% and 6%, respectively). In fact, EGb contains 6% terpenoids (in which 3.1% are ginkgolides A, B, C, and J and 2.9% is bilobalide), 24% flavonoid glycosides, and 5% to 10% organic acids [10]. The flavonoids act as free radical scavenging, whereas terpenes lactones protect mitochondrial membranes from free radical damage [11]. EGb has been described to have antioxidant properties playing an important role as a free radical scavenger [12]. It has demonstrated that the antioxidant activity, as a “radical scavenger”, is due to its superoxide dismutase-like activity that enables it to scavenge hydroxyl radicals [13]. Ginkgo biloba also has the capacity to regulate the oxidative stress. The levels of glutathione, malondialdehyde, superoxide dismutase and nitric oxide, increased after a treatment with EGb [14]. These properties determine beneficial effects in neurodegenerative diseases as Alzheimer [15,16] or Parkinson [17,18]. However, the mechanism of the action of EGb protection against oxidative stress-induced apoptosis remains to be fully elucidated.

The evidence mentioned above prompted us to explore the protective effect of EGb against oxidative stress-induced apoptosis in neuroblastoma cells. Our data suggested that this extract could act as cellular scavengers against induced oxidative stress blocking the onset of molecular apoptotic pathway.

## 2. Materials and Methods 

### 2.1. Cell Culture and Chemicals

Human neuroblastoma cell line, SK-N-BE(2) (CRL-2271, ATCC^®^, LGC Standards S.r.l., Milan Italy) were cultured at 37 °C in a 5% CO_2_ humidified incubator in either RPMI-1640 medium (Euroclone spa, 20016 Pero, MI) supplemented with 10% fetal bovine serum (FBS), glutamine (2 mM), sodium pyruvate and antibiotics (0.02 mg/mL streptomycin and 0.02 IU/mL penicillin).

*Ginkgo biloba* L. extract EGb 761 (EGb) was a gift from Schwabe (Schwabe Pharma Italia Srl, Egna, Italy). EGb stock solution contained 250 mg/mL of extract was dissolved in dimethyl sulfoxide (DMSO). Hydrogen peroxide (H_2_O_2_,) (Sigma-Aldrich, St. Louis, MI, USA) was used as oxygen stress inducer.

### 2.2. Cell Proliferation Assay

For each experiment, approximately 1.5 × 10^5^ cells/well in 6-well plates were plated and treated as described and untreated cells were used as control. To identify the H_2_O_2_ concentration able to determine about 50% of viability decrease, SK-N-BE cells were treated with 25, 50, 75, and 100 mM of H_2_O_2_ for 24 h. When specified cells were treated with 25 μg/mL EGb for 24 h, the medium was replaced before H_2_O_2_ treatment.

To evaluate the effect of EGb on cell viability, cells were treated with 10, 25, and 50 μg/mL for 24 h. To estimate the protective effect of EGb cells were treated for 24 h with 25 μg/mL of EGb, then insulted with 75 µM of H_2_O_2_ for additional 24 h. EGb was dissolved in DMSO. Untreated samples were exposed to 0.1% DMSO and were used as control.

For each experiment after treatment cells were collected and counted with Trypan Blue solution. (T6146, Sigma-Aldrich, St. Louis, MI, USA).

All the experiments were performed in triplicate. Data are expressed as the mean ± SD.

### 2.3. Propidium Iodide and DAPI Staining Assay

In 6-well plates approximately 1.5 × 10^5^ cells/well were plated and treated with EGb and H_2_O_2_ as previously described. After treatment cells were stained with 10 mg/mL of Propidium Iodide (PI) (Bioshop, Burlington, ON L7L 6A4, Canada) and DAPI (4′,6-diamidine-2′-phenyl indole dihydro chloride, Roche, Mannheim, Germany). Representative images were taken using fluorescent microscope (DMI8, Leica, Instruments, Germany) and florescence was quantified using Leica Application Suite X software (Leica, Milan, Italy). All the experiments were performed in triplicate. Data are expressed as the mean ± SD.

### 2.4. Mitochondria Membrane Potential Measurement 

Mitochondria membrane potentials (MMP) were measured by JC-10 (Sigma-Aldrich, St. Louis, MI, USA) following the manufacturer’s instructions. Loss of MMP was indicated by a progressive JC-10 dislocation from mitochondria to the cytosol. Cells were photographed using fluorescent microscope (DMI8, Leica, Instruments, Germany). Red (540/570 nm) and green (485/534 nm) florescence was quantified by Leica Application Suite X (LAS X) (Leica, Milan, Italy). All the experiments were performed in triplicate. Data are expressed as the mean ± SD.

### 2.5. RNA Extraction and q-PCR

RNA extraction and q-PCR were essentially performed as previously described [19]. Brefly, Total RNA was isolated from each sample with Trizol (Thermo Fisher Scientific, Waltham, MA USA), as indicated by manufacturer. For each sample to analyse, cDNA was than obtained starting from 200 ng of total RNA using High Capacity cDNA Reverse Transcription Kit (Applied Biosystem, Thermo Fisher Scientific, Waltham, MA USA). The described selected genes using gene specific primers

BAX: Forward 5′-TTTGCTTCAGGGTTTCATCCA-3′: Reverse 5′- CTCCATGTTACTGTCCAGTTCGT-3′; BCL- 2: Forward 5′-GTTCCCTTTCCTTCCATCC-3′; Reverse 5′-TAGCCAGTCCAGAGGTGAG-3′; p53: Forward 5’-TCTGTCCCTTCCCAGAAAACC-3’; Reverse 5’-CAAGAAGCCCAGACGGAAAC-3′; GAPDH: Forward 5′-CAAGGCTGTGGGCAAGGT-3′; Reverse 5′-GGAA GGCCATGCCAGTGA-3’.

All primers were selected using a specific software (Primer express 2.0, Applied Biosystems, Foster city, CA, USA) and all of them specifically covered the exon-exon junctions. The analysis of gene expression was done as described in [20] and GAPDH gene was used as internal control. qPCRs were done using the 7900 HT Real Time PCR (Applied Biosystem) and for each experimental condition a triplicate was performed. Data obtained are expressed as the mean ± SD.

### 2.6. Western Blot

For each experimental condition and from each sample total, protein extracts were obtained, as described in [19]. For the analysis, 20 μg of each sample were loaded on Tris–glycine gradient gels (4% to 15% gels (Bio-Rad Laboratories, Inc., Hercules, CA, USA) and separated at 100 V. To probe proteins with specific primary antibodies antibodies, they were transferred to PVDF membranes (Biorad Laboratories, Inc., Hercules, CA, USA). All the secondary antibodies used were horseradish peroxidase conjugated. All the antibodies were used as indicated by manufacturer. The following primary antibodies were used for Western blot: PARP (Cell Signaling, #9542), BCL-2 (Abcam, ab182858), BAX (Santa Cruz Biotechnology, sc-493), Acetyl-p53 Lys382 (Cell Signaling, #9542). As the internal control we used β-Actin (Cell Signaling, #3700), which was used as the loading control. To detect protein levels, Clarity western ECL (Bio-Rad Laboratories, Inc., Hercules, CA, USA) was used. The quantization was then obtained using ImageJ software vJ1, an open source tool. For each experimental condition a triplicate was performed, and results are expressed as the mean ± SD.

### 2.7. Statistical Analysis

To perform calculations on sample size the on line available software GPower was used. Sample size was determined using as parameters: 1 − β = 0.80, α = 5%. For each experiment, statistical analysis was done using Graph Pad Prism 6.0 (GraphPad Software, San Diego, CA, USA) to analyse the significance of the differences between control and treatments. We evaluated the differences among means applying the one-way ANOVA. Bonferroni’s multiple comparison test with Bonferroni post hoc correction was used to analyse the differences of each treatment respect to the control.

Statistically significant difference compared to DMSO treated cells are: * *p* ≤ 0.05, ** *p* ≤ 0.01, *** *p* ≤ 0.001, **** *p* ≤ 0.0001.

## 3. Results

### 3.1. EGb Protects SK-N-BE Cells Against Oxidative Stress Induced Apoptotic Cell Death

**Figure 1:**
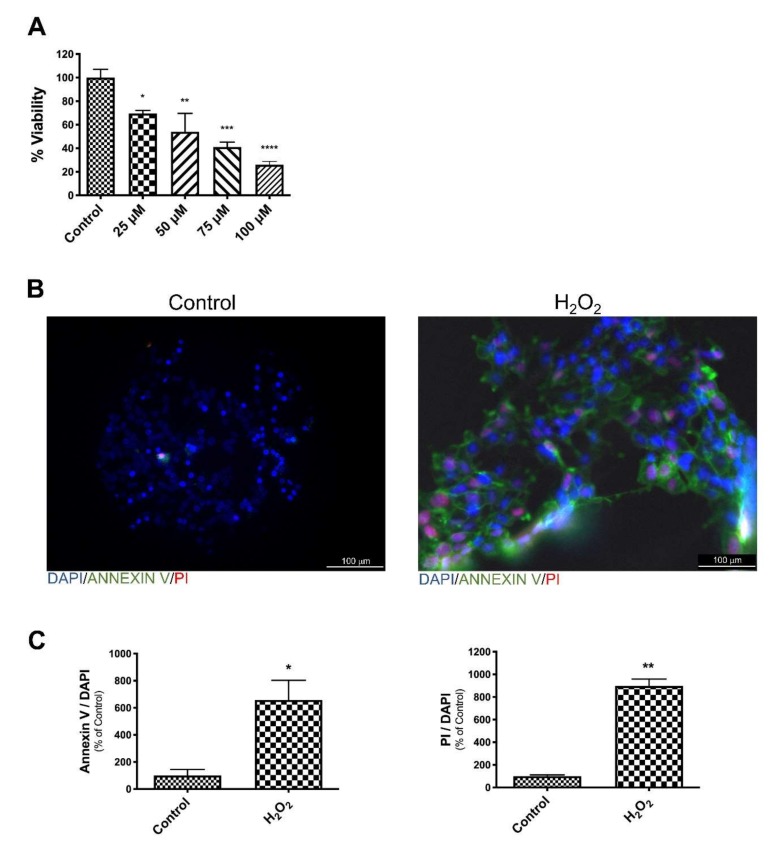
H_2_O_2_ affects SK-N-BE cell viability. Cell viability decrease after treatments with different concentration of H_2_O_2_ (**A**). representative images of DAPI, Annexin V-FITC and PI triple fluorescence staining showing cellular apoptosis after H_2_O_2_ treatment. DAPI: blue; AnnexinV: green; PI: red (**B**). Histograms reports quantification of fluorescence of DAPI, Annexin V, and PI (**C**). The bars represent ± the average ± SD of independent experiments (*n* = 3). Statistically significant difference compared to control cells: * *p* ≤ 0.05, ** *p* ≤ 0.01, *** *p* ≤ 0.001, **** *p* ≤ 0.0001.

We first determined, in a dose-response curve at 24 h, the amount of H_2_O_2_ that had lethal effect on SK-N-BE human neuroblastoma cells. Oxidative stress induced cell death was around 50% when cells were treated with 75 µM H_2_O_2_ (Figure 1A). To verify if 75 µM H_2_O_2_ was able to induce apoptosis on SK-N-BE cells, untreated and H_2_O_2_ treated cells, without fixation and permeabilization, were stained with AnnexinV-FITC and PI. The analysis by fluorescence microscopy confirmed that H_2_O_2_, at this concentration, induced apoptosis (Figure 1B). Indeed, a strong increased number of stained AnnexinV-FITC and PI cells were present when cells were treated with H_2_O_2_ (Figure 1C).

**Figure 2:**
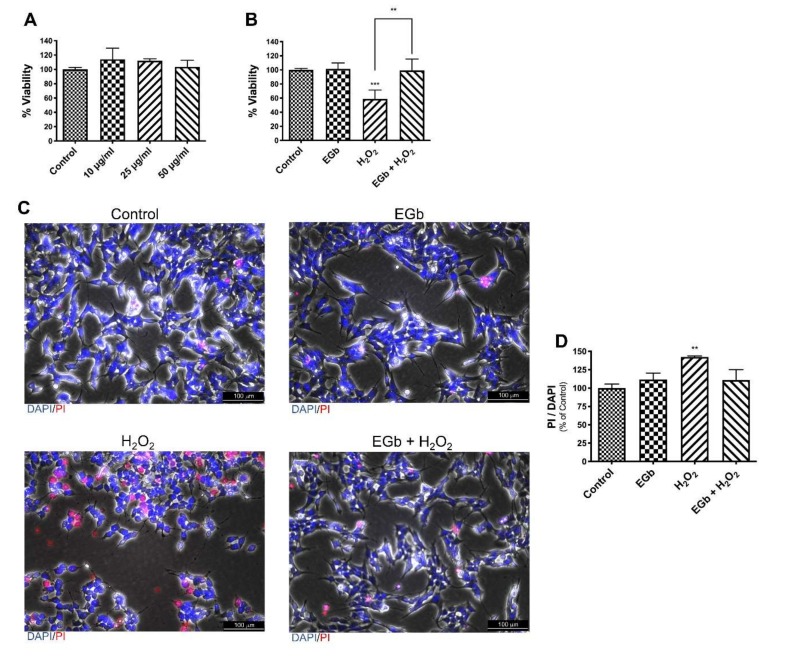
Effects of EGb on cell viability. Cell viability after treatments with different concentration of EGb (**A**). Cell viability after treatments with 25 mg/mL of EGb, 75 mM of H_2_O_2_ or a combination of them (**B**). Cells treated with DMSO were used as control. Fluorescent microscopic image of DAPI/PI stained cells; DAPI: blue; PI: red (**C**). Histogram reports quantification of fluorescence of DAPI and PI (**D**). The bars represent ± the average ± SD of independent experiments (*n* = 3). Statistically significant difference compared to control cells: ** *p* ≤ 0.01, *** *p* ≤ 0.005.

To ascertain that EGb did not induce cell death, SK-N-BE cells were treated with various concentrations of EGb for 24 h. Results showed that EGb at all used concentrations did not reduce cell viability (Figure 2A).

To determine whether EGb played a role in protecting SK-N-BE from H_2_O_2_ -induced cell death, cells were pre-treated for 24 h with EGb (25 μg/mL) and then challenged with H_2_O_2_ (75 µM) for the following 24 h. Analysis of cell vitality revealed that the oxidant sensitivity of SK-N-BE cells was completely reverted by pre-treatment with EGb (Figure 2B). Concomitant addition of EGb and H_2_O_2_ or addition of EGb 24 h later H_2_O_2_ treatment did not result in a reversion of lethality (Figure S1). Results were confirmed by analysis with PI and DAPI staining, as shown by fluorescence microscopy analysis. Indeed, a comparable number of the PI positive cells were present in untreated, EGb treated and EGb-H_2_O_2_ treated cells, where a higher number were present in presence of H_2_O_2_ alone (Figure 2C,D).

### 3.2. EGb Protects SK-N-BE Cells Against Oxidative Stress Induced Apoptosis

**Figure 3:**
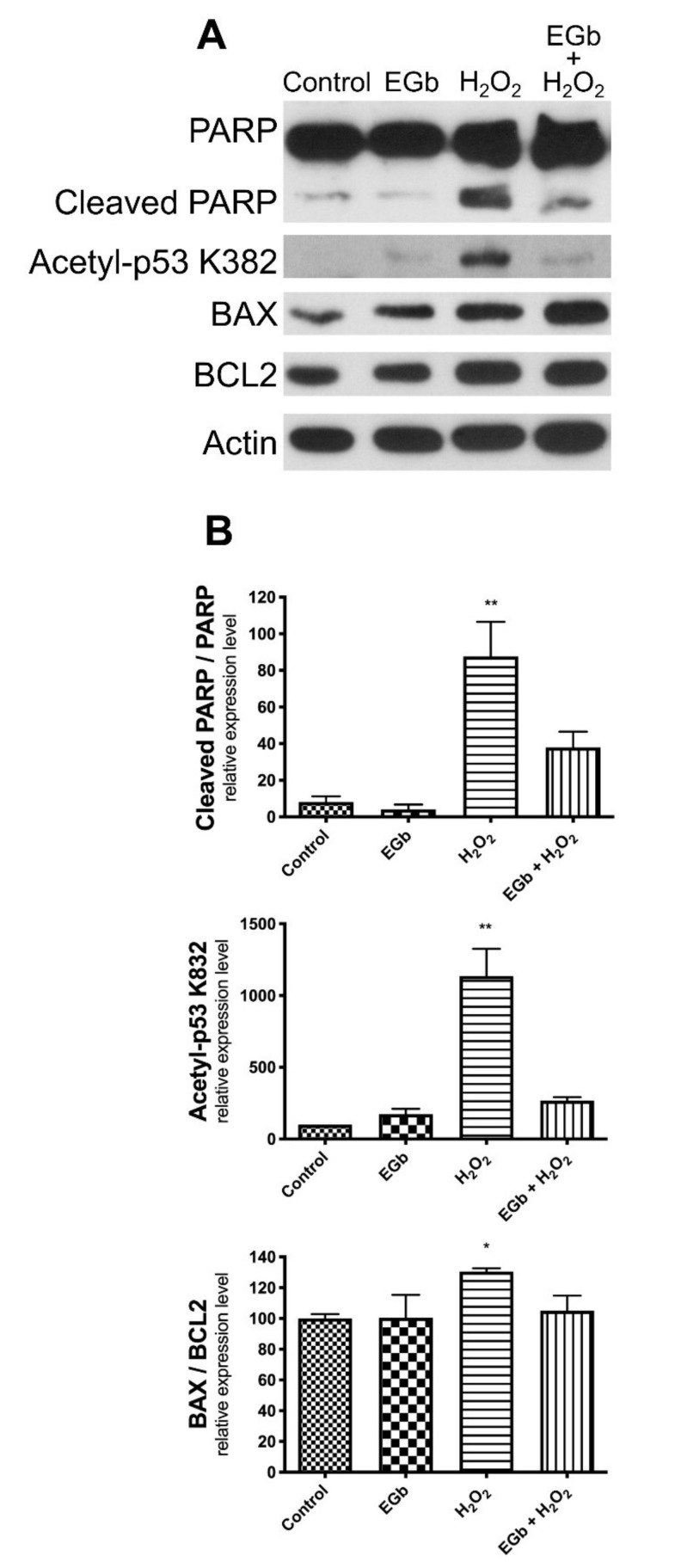
EGb protects SK-N-BE from apoptosis. Western blot analysis of protein expression of cleaved PARP, Acetylated-p53/K382, BAX and Bcl2 in SK-N-BE cells after treatments (**A**). expression of Acetylated-p53/K382, cleaved PARP and BAX/Bcl2 ratio normalized expression is reported in the histograms (**B**). β-Actin was used as loading control. The bars represent ± the average ± SD of independent experiments (*n* = 3). Statistically significant difference compared to control cells: * *p* ≤ 0.05, ** *p* ≤ 0.01. Cells treated with DMSO were used as control.

To confirm that EGb could protect cells against apoptotic cell death induced by oxidative stress, we first analysed the presence of poly(ADP-ribose) polymerase (PARP) cleavage, an hallmark of apoptosis. As expected, PARP cleavage increased after H_2_O_2_ treatment, although the cells were completely protected from oxidative stress-induced apoptosis in presence of EGb (Figure 3A,B). Then, to study the protective mechanism of EGb against oxidative stress-induced apoptosis, we investigated the molecular signalling pathway involved in the apoptotic cell death analysing p53 expression.

The tumor suppressor protein p53, modulating cell homeostasis, has a determinant role in cell fate. 

Oxidative stress, leading to post-translational modifications of p53, allows it to regulate genes that can activate cell survival or cell death processes [21]. 

Gene expression analysis, by q-PCR, revealed that p53 was not modulated by oxidative stress as well as by EGb (Figure S2). It is known that increased p53-acetylation at lysine 382 (K382) promotes p53-dependent pro-apoptotic activity in cancer cells [22]. Thus, we analysed by western blot analysis whether these post-translational modifications of p53 could account for the apoptotic reduction observed in presence of EGb. Results clearly demonstrate that K382 acetyl-p53 was strongly increased following H_2_O_2_ insult, however p53-acetylation was inhibited in presence of EGb (Figure 3A,B).

Oxidative stress activates the mitochondrial intrinsic pathway of apoptosis [23]. p53, interacting with members of the Bcl-2 family, directly participating to the activation of the intrinsic apoptosis pathway [24]. We focused our attention on the ratio between two members of the Bcl-2 family, BAX and BCL-2, which are markers of cell susceptibility to intrinsic apoptosis. Protein expression analysis evidenced an increased BAX/Bcl-2 ratio in H_2_O_2_ treated SK-N-BE while pre-treatment with EGb restored a normal ratio (Figure 3A,B). These results were also confirmed by gene expression analysis, by q-PCR of the corresponding genes (Figure S1).

### 3.3. EGb Mitigated the H_2_O_2_ Induced Decrease in Mitochondrial Membrane Potential

**Figure 4:**
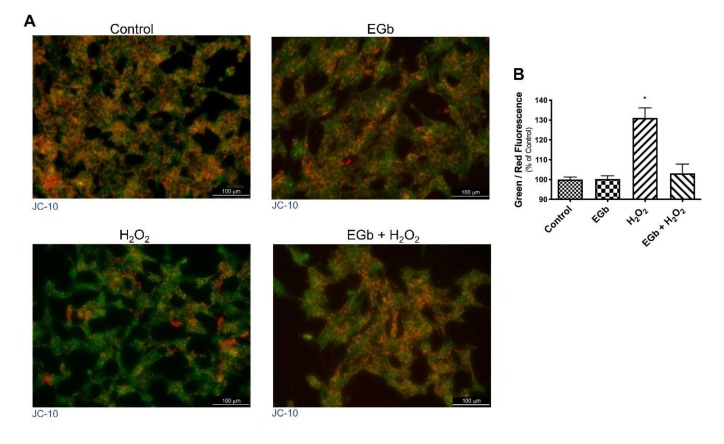
EGb reduce the decrease of mitochondrial membrane potential. Fluorescence analysis of mitochondria in control or EGb treated cells with or without H_2_O_2_ (**A**). Histogram reports quantification of fluorescence of Red (540/570 nm) and green (485/534 nm) (**B**). The bars represent ± the average ± SD of independent experiments (*n* = 3). Statistically significant difference compared to control cells: * *p* ≤ 0.05). Cells treated with DMSO were used as control.

Increased BAX/Bcl-2 ratio suggested that mitochondria are involved in apoptosis. Indeed, it is well known that, during intrinsic apoptosis, the mitochondrial membrane potential (MMP) collapses, triggering other downstream events in the apoptotic cascade. Thus, we investigated by JC-10 assay the change of MMP following H_2_O_2_ or EGb treatment of SK-N-BE cells. Results showed that untreated cells displayed intact, well-polarized mitochondria marked by a red punctate fluorescence. On contrary, H_2_O_2_ treated cells showed a reduction of the red fluorescence and an increase of the green one, indicating loss of MPM because of the progressive JC-10 dislocation from mitochondria to the cytosol. On the contrary, EGb treatment restored the fluorescence to the values of untreated cells. (Figure 4).

### 3.4. EGb Exhibits Intracellular Anti-Apoptotic Effect

**Figure 5:**
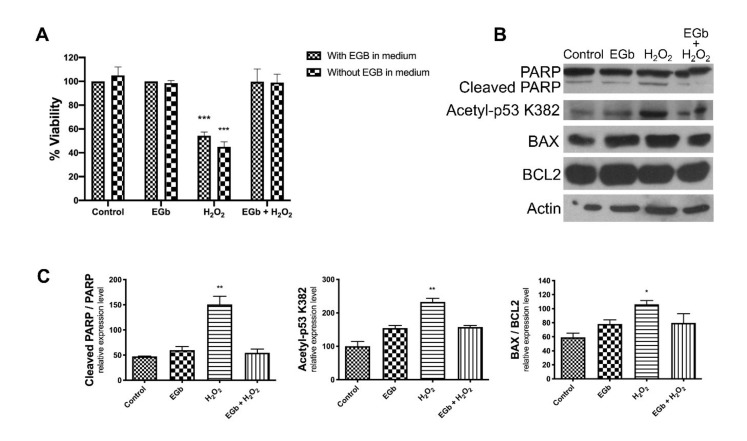
Intracellular effect of EGb. Cell viability analysis of SK-N-BE with or without EGb in the medium after H_2_O_2_ oxidative insult (**A**). Protein expression analysis of cleaved PARP, Acetylated-p53/K382, BAX and Bcl2 in SK-N-BE cells after treatments by Western blot analysis (**B**). Histograms report the expression of Acetylated-p53/K382, cleaved PARP and BAX/Bcl2 ratio normalized expression (**C**). β-Actin was used as loading control. The bars represent ± the average ± SD of independent experiments (*n* = 3). Statistically significant difference compared to control cells: * *p* ≤ 0.05, ** *p* ≤ 0.01, *** *p* ≤ 0.005). Cells treated with DMSO were used as control.

To verify whether EGb acts as antioxidant into cells, or if it was able to directly scavenge H_2_O_2_ in the culture medium, SK-N-BE cells were treated for 24 h with EGb, then the culture medium was replaced and cells were challenged with H_2_O_2_. Results showed that that EGb determined antioxidant protection on cell viability independently by its presence in the culture medium. In fact, pre-treatment with EGb was per se sufficient to attenuate the H_2_O_2_-induced cell death in SK-N-BE cells (Figure 5A). Moreover, in these conditions we observed a reduced cleavage of PARP protein, a reduced amount of K382 acetyl-p53 and a reduced BAX/Bcl-2 ratio (Figure 5B,C), confirming that EGb was able to protect SNKBE cells by apoptotic cell death exerting an intracellular antioxidant action.

## 4. Discussion

The incidence of neurological disorders—the most dreaded maladies of older people—are expected to increase over the next few decades due to prolonged life expectancy [25]. To date, more than 1 in 10 individuals over 65 years are affected by neurodegenerative diseases and the numbers will continue to increase with age. Until now, no effective treatments have been described to cure these diseases and the costs for their management represent one of the leading medical and societal challenges faced by society [26]. For these reasonsm recent investigations have been focused to understanding their pathogenesis and to the development of novel therapeutics. *Ginkgo biloba*, a plant that has been used for thousands of years, is considered one of the more promising natural drugs. The extracts, obtained from *Ginkgo biloba* leaves, have been recently used also in clinical studies.

Most of the studies on the EGb concern its neuroprotective effects, against ageing. Standardized Ginkgo biloba extracts (EGb) have been used for treatment and prevention of different neurological disorders as Alzheimer’s disease [15,16], Parkinson’s disease [17,18], cerebral vascular deficit, and dementia [27,28].

Indeed, the brain is especially sensitive to the effects of ageing. This tissue being primarily composed of postmitotic cells—neurons and oligodendrocytes—is more vulnerable than proliferating cells to macromolecular damages, especially to DNA [29]. DNA damages in neurons accumulate from development throughout life. To escape this process, postmitotic neurons adopt selective mechanisms aimed to specifically repair genes actively transcribed [30].

Oxidative stress is one of the main causes of neural damage. EGb exerts neuroprotective action mainly acting as free radical–scavenger In fact, EGb is able to reduce the endogenous and the induced levels of ROS [31]. Moreover, EGb can directly upregulate antioxidant enzymes such as superoxide dismutase and catalase [32]. This activity is linked to the chemical structure of the flavonoids that allow to not only react and directly scavenge the hydroxyl radicals, but also to inhibit the formation of new hydroxyl radicals [33]. It is well known that oxidative stress determines the activation of the apoptotic processes, thus playing a pivotal role in most of neurological diseases. EGb can act on multiple cellular pathways with the final goal to balance the existing apoptotic machinery. In fact, EGb prevents mitochondrial membrane damage reducing the release of cytochrome c from the mitochondria, upregulates the antiapoptotic protein Bcl-2-and inhibit PARP cleavage [34]. 

The neuroprotective effects of EGb 761 has been reported in different neuronal cell lines in which it acts by inhibiting oxidative stress induced apoptosis [35,36,37] or the activation of mitochondrial intrinsic apoptosis [35,36]. A recent in vivo study reported that Egb761 protected from brain injury by suppressing neuronal apoptosis [9]. Moreover, some studies reported the protective effect of Ginkgo biloba extract in people affected by neurodegenerative diseases [38,39]. 

In this study we analysed the protective effect of EGb on oxidative stress-induced apoptosis in SK-N-BE cells with the aim to unravel the molecular pathway in which EGb acts as antioxidant. Human neuroblastoma cell line N-type have neuronal morphology [40] and have been commonly used as model for research in neuroscience and in particular in studies related to oxidative stress and neurodegenerative diseases [41,42,43,44]. 

Our results demonstrated that the standardized extract EGb 761 significantly protected neuroblastoma cells from oxidative stress blocking apoptosis in a p53-dependent pathway. Interestingly, according to previous studies we found that EGB was able to inhibit p53 acetylation at lysine 382. It is known that p53 activity depends on the acetylation of specific lysines [45]. In addition, the acetylation of the C-terminal K382 lysine is crucial for p53 activation [46] since it results in the activation of PUMA promoter—a member of Bcl-2 family [47]. PUMA, promoting BAX multimerization and mitochondrial translocation, induces apoptosis [48]. Accordingly, our results show that EGb protects against mitochondrial membranes depolarization with a consequent reduction of BAX/Bcl-2 ratio. These results were supported by reduction of PARP cleavage with increased viability. 

Previous studies reported that *Gingko biloba* extracts in cancer cells is able to induce apoptosis in a p53-dependent pathway by increasing the levels of p53 acetylation that, in turn, determines cell cycle arrest and apoptosis. On the contrary, our results demonstrated that the standardized extract EGb 761 significantly protected neuroblastoma cells from oxidative stress blocking apoptosis in a p53-dependent pathway. These results claim the different activity of EGb when used as neuroprotective or as anticancer drug [49].

## 5. Conclusions

Neurodegenerative disorders include a range of conditions that share common degenerative pathways, although they manifest with clinical differences. Increased oxidative stress has been described in almost all neurodegenerative disorders. In neurons, imbalance between the accumulatin of free radicals and antioxidant defences seems to be the link between cell death and progression of neurodegenerative diseases. Oxidative stress can trigger apoptosis in neuronal cells and excessive death of one or more populations of neurons, resulting in a neurodegenerative disease [50]. 

Our data suggest that EGb 761 could be considered an active antioxidant nutraceutical to be used for the prevention and treatment of neurodegenerative diseases.

Our data suggest that EGb 761, blocking the onset of p53-dependent apoptotic pathway induced by oxidative stress, could be considered as antioxidant nutraceutical to be potentially used for the prevention and treatment of neurodegenerative diseases. This hypothesis could be strengthened with a larger number of randomized clinical trials.

## Supplementary Materials

The following are available online at https://www.mdpi.com/2076-3921/9/4/279/s1, **Figure S1**: Cell viability after treatments with 25 μg/mL of EGb, 75 μM of H_2_O_2_ or a combination of them. In Egb + H_2_O_2_ (1) cells were treated for 24 h with EGb and then 24 h with H_2_O_2_. In EGb + H_2_O_2_ (2) cells were treated with EGb and H_2_O_2_ concurrent for 24 h. In EGb + H_2_O_2_ (3) cells were treated for 24 h with H_2_O_2_ and then with EGb for additional 24 h. The bars represent ± the average ± SD of independent experiments (*n* = 3). Statistically significant difference compared to control cells: ** *p* ≤ 0.01, *** *p* ≤ 0.005, **** *p* ≤ 0.001. Cells treated with DMSO were used as control. **Figure S2**: q-PCR analysis. Quantitative analysis of mRNA expression levels of p53, BAX and Bcl-2 in SK-N-BE cells after treatments. Histograms report the expression of p53 (**A**) and BAX/Bcl2 ratio (**B**). The bars represent ± the average ± SD of independent experiments (*n* = 3). Statistically significant difference compared to untreated cells: ** *p* ≤ 0.01.
