# Supplementary material for: Ginkgo biloba Prevents Oxidative Stress-Induced Apoptosis Blocking p53 Activation in Neuroblastoma Cells
Source: Antioxidants (Basel). 2020 Mar 26;9(4):279. doi: 10.3390/antiox9040279 (PMC7222193; doi:10.3390/antiox9040279)
Supplement: Supplementary file 1 [file antioxidants-09-00279-s001.pdf]

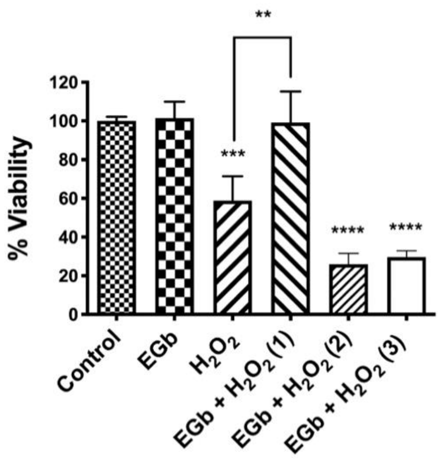

Figure S1. Cell viability after treatments with 25 g/mL of EGb, 75 M of H_2O_2 or a combination of them. In Egb + H_2O_2 (1) cells were treated for 24 h with EGb and then 24 h with H_2O_2. In EGb + H_2O_2 (2) cells were treated with EGb and H_2O_2 concurrent for 24 h. In EGb + H_2O_2 (3) cells were treated for 24 h with H_2O_2 and then with EGb for additional 24 h. The bars represent ± the average ± SD of independent experiments (n = 3). Statistically significant difference compared to control cells: **$p \leq 0.01$, ***$p \leq 0.005$, ****$p \leq 0.001$. Cells treated with DMSO were used as control.

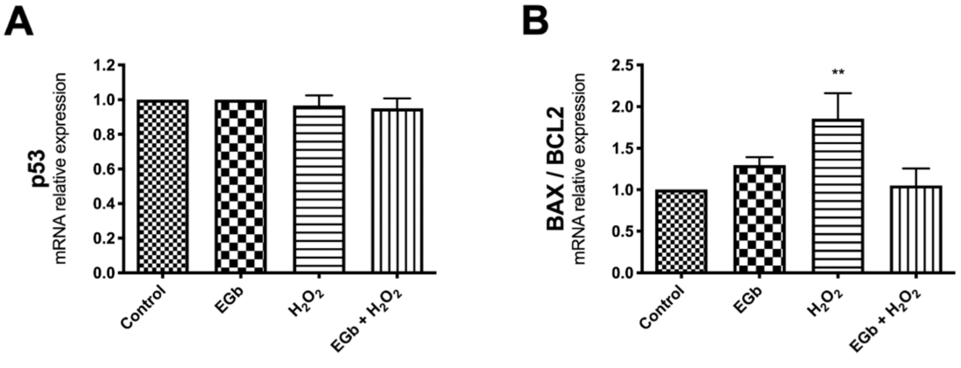

Figure S2. **q-PCR analysis**. Quantitative analysis of mRNA expression levels of p53, BAX and Bcl-2 in SK-N-BE cells after treatments. Histograms report the expression of p53 (**A**) and BAX/Bcl2 ratio (**B**). The bars represent ± the average ± SD of independent experiments (n = 3). Statistically significant difference compared to control cells: **p ≤ 0.01. Cells treated with DMSO were used as control.
